# Supplementary material for: Characterizing cell-type spatial relationships across length scales in spatially resolved omics data
Source: Nat Commun. 2025 Jan 3;16:350. doi: 10.1038/s41467-024-55700-1 (PMC11699133; doi:10.1038/s41467-024-55700-1)
Supplement: Supplementary file 3 — Supplementary Software 1 [file 41467_2024_55700_MOESM3_ESM.zip › CRAWDAD/docs/tutorial.html]

tutorial


# Installation

```
## for installation once the GitHub repo is public

require(remotes)
remotes::install_github('JEFworks-Lab/CRAWDAD')
```

```
## for bioconductor (once its been submitted and approved)

if(!requireNamespace("BiocManager", quietly = TRUE))
    install.packages("BiocManager")
BiocManager::install("CRAWDAD")
```

```
library(crawdad)
library(dplyr)
```

# Tutorial

```
ncores <- 2
```

## Make simulated dataset

```
data(sim)

## visualize
plt <- crawdad::vizAllClusters(cells = sim,
                               coms = sim$type,
                               title = "sim",
                               axisAdj = 1, s = 6, a = 0.5) +
  ggplot2::guides(colour = ggplot2::guide_legend(override.aes = list(size=2), ncol = 1))
plt
```

```
## convert to SP
cells <- crawdad::toSP(pos = sim[,c("x", "y")],
                        celltypes = sim$type)
```

```
## Warning: 'celltypes' does not have levels. Creating levels from values

## creating `sp::SpatialPointsDataFrame`
```

```
cells
```

Convert the data.frame of cells to an
`sp::SpatialPointsDataFrame` object. This is because CRAWDAD
builds upon the `sf` library in R.

## Make shuffled background

`CRAWDAD` identifies cell type spatial relationships by
comparing cell type organizational patterns in the real data to a set of
null distributions, which are a datasets in which cell labels have been
shuffled at different scales, or resolutions. We can generate this list
of shuffled datasets with the following code:

```
oldw <- getOption("warn")
options(warn = -1)

## generate background
shuffle.list <- crawdad::makeShuffledCells(cells,
                          resolutions = c(100, 200, 500, 800, 1000),
                          perms = 1,
                          ncores = ncores,
                          seed = 1,
                          verbose = TRUE)
```

```
## shuffling permutation 1 using seed 1

## 100 unit resolution

## 100 tiles to shuffle...

## shuffling permutation 1 using seed 1

## 200 unit resolution

## 25 tiles to shuffle...

## shuffling permutation 1 using seed 1

## 500 unit resolution

## 4 tiles to shuffle...

## shuffling permutation 1 using seed 1

## 800 unit resolution

## 4 tiles to shuffle...

## shuffling permutation 1 using seed 1

## 1000 unit resolution

## 1 tiles to shuffle...

## Time was 0.19 mins
```

```
options(warn = oldw)
```

## Run pairwise analysis

We can identify trends that describe spatial relationships between
pairwise combinations of cell types in our data. `dist`
refers to the distance at which neighbor cells are defined. In this
example, we assess if the neighbors of each cell type are enriched or
depleted in cells of another given cell type compared to each shuffled
resolution of the data.

```
oldw <- getOption("warn")
options(warn = -1)

## find trends, passing background as parameter
results <- crawdad::findTrends(cells,
                        dist = 100,
                        shuffle.list = shuffle.list,
                        ncores = ncores,
                        verbose = TRUE)
```

```
## Evaluating significance for each cell type

## using neighbor distance of 100

## Calculating for pairwise combinations

## A

## B

## C

## D

## Time was 0.75 mins
```

```
options(warn = oldw)
```

## Visualizing trends

We can now visualize trends for different pairwise cell type
combinations using the following code:

By default, `vizTrends()` is setup to facet wrap reference
and neighbor cell type trends. Reference cell types are column headers
and neighbor cell types are row headers.

```
plt <- crawdad::vizTrends(dat = results)
```

```
## results detected to be a list. Melting to data.frame.
```

```
plt
```

```
## because the output is a `ggplot2` object, we can perform additional manipulations, like log-transformation of the x-axis:
plt <- crawdad::vizTrends(dat = results) +
  ggplot2::scale_x_log10()
```

```
## results detected to be a list. Melting to data.frame.
```

```
plt
```

We can also turn off the facet wrapping, and then choose to plot
multiple trend lines in the same plot using the `id` column
(or another column) in the melted results data.frame. This can be useful
if we want to compare the overlay of two trends:

```
## melt the results into a data.frame
dat <- crawdad::meltResultsList(results)

## select different trend combinations
d1 <- dat[dat$reference == "A" & dat$neighbor == "A",]
plt <- crawdad::vizTrends(dat = d1) +
  ggplot2::scale_x_log10()
plt
```

```
d2 <- dat[dat$reference == "A" & dat$neighbor == "B",]
plt <- crawdad::vizTrends(dat = d2) +
  ggplot2::scale_x_log10()
plt
```

```
## combine the trends into one data.frame, and have the "id" column label the combo, plot both lines on same plot
## turn off the facet wrap so just coloring the two trends, which are labeled using the "id" column

d1$id <- "A vs A"
d2$id <- "A vs B"

d <- dplyr::bind_rows(list(d1, d2))

plt <- crawdad::vizTrends(dat = d, facet = FALSE) +
  ggplot2::scale_x_log10()
plt
```

Instead, of plotting trends, we can also visualize the Z scores
across resolutions as heatmaps:

```
plt <- crawdad::vizTrends.heatmap(dat = results, annotation = TRUE,
                                  z_limit = 20, # Z score limits (default +/- 20). Winsorize values to these limits
                                  sig.thresh = 1.96, # Z score significance threshold (default: 1.96). Non-significant values < 1.96 and > -1.96 colored white
                                  ncols = 4 # specify number of columns in facet wrap (default: 4)
                                  )
```

```
## results detected to be a list. Melting to data.frame.
```

```
plt
```

And likewise, we can also choose to visualize specific cell type. For
example, just reference cell type A and its neighbor interactions with
cell types A and B:

```
## melt the results into a data.frame
dat <- crawdad::meltResultsList(results)

## select different trend combinations
dat <- dat[dat$reference == "A" & dat$neighbor %in% c("A", "B"),]

plt <- crawdad::vizTrends.heatmap(dat = dat)
plt
```

## Filtering trends

We can filter down the different cell type interactions to those that
are significantly colocalized (positive significant Z score for at least
one resolution), those that are significantly separated (negative
significant Z score for at least one resolution), or those that shift
between being colocalized and separated at different resolutions (at
least one positive and negative significant Z score for different
resolutions).

```
results.coloc <- crawdad::filterCoTrends(results = results, alpha = 0.05)
results.sep <- crawdad::filterSepTrends(results = results, alpha = 0.05)
results.change <- crawdad::filterChangeTrends(results = results, alpha = 0.05)
```

```
plt <- crawdad::vizTrends.heatmap(dat = results.coloc, title = "Significant colocalized cell types")
```

```
## results detected to be a list. Melting to data.frame.
```

```
plt
```

```
plt <- crawdad::vizTrends.heatmap(dat = results.sep, title = "Significant separated cell types")
```

```
## results detected to be a list. Melting to data.frame.
```

```
plt
```

## Defining subsets

We can also further subdivide cell types into subsets by looking for
cell types whose neighbors are enriched in another particular cell type.
We can do this by performing a binomial test to see if a cell’s
neighbors are significantly enriched in a particular cell type compared
to the overall probability of observing that particular cell type.

For this dataset, we define a neighbor distance of 100 to
characterize cells into subsets.

First, we generate a probability matrix where rows are each cell in
the dataset, columns are the cell type labels, and values are the
probability of each cell being enriched in neighbors of a given cell
type.

```
oldw <- getOption("warn")
options(warn = -1)

binomMat <- crawdad::binomialTestMatrix(cells,
                               neigh.dist = 100,
                               ncores = ncores,
                               verbose = TRUE)
```

```
## Binomial test for each cell testing if it is enriched in neighbors of a given cell type based on distance of 100

## Performing tests...

## Time to compute was 0.44mins
```

```
head(binomMat)

options(warn = oldw)
```

We can now assign cells to subsets based on how significant their
neighbors are enriched with a given cell type. For finding subsets of
cells that are enriched with a given cell type, we can set the
`sub.type` to “near”, with a `sub.thresh` of 0.05
and sub-categorize cells of a given cell type if they are enriched with
another cell type at a p-value of 0.05 or less.

Conversely, we can also set `sub.type` to “away” and
sub-categorize cells of a given cell type if they are depleted with a
given cell type. In this case, setting the `sub.thresh` to
above 0.5 would be advised, because in this way, cells above a p-value
of 0.5 in terms of testing for enrichment would be selected. In other
words, only the cells that couldn’t even pass a p-value cutoff of 0.5
would be selected for, and these would be expected to be very much
depleted or separated from the neighbor cell type they are being
compared to.

In this tutorial, we will define subsets of cell types whose
neighbors are enriched in another cell type, where neighbors were
defined using a distance of 100 and a p-value threshold of 0.05:

```
oldw <- getOption("warn")
options(warn = -1)

subset.list <- crawdad::selectSubsets(binomMat,
                             cells$celltypes,
                             sub.type = "near",
                             sub.thresh = 0.05,
                             ncores = ncores,
                             verbose = TRUE)
```

```
## computing subsets for A_near_A
## computing subsets for B_near_A
## computing subsets for C_near_A
## computing subsets for D_near_A
## computing subsets for A_near_B
## computing subsets for B_near_B
## computing subsets for C_near_B
## computing subsets for D_near_B

## computing subsets for A_near_C
## computing subsets for B_near_C
## computing subsets for C_near_C
## computing subsets for D_near_C
## computing subsets for A_near_D
## computing subsets for B_near_D
## computing subsets for C_near_D
## computing subsets for D_near_D

## Time to compute was 0.03mins
```

```
subset.list["C_near_B"]

options(warn = oldw)
```

For example, these cell ids are the cells of cell type “C” that are
enriched with cell type “B”.

## Visualize the subsets

To visualize how the subsets are defined, let’s first look at just
the “C” cells and the “B” cells

```
plt <- crawdad::vizAllClusters(cells = cells,
                               coms = cells$celltypes,
                               ofInterest = c("B", "C"), ## just color these cell types
                               title = "B and C cells",
                               axisAdj = 1, s = 6, a = 0.5) +
  ggplot2::guides(colour = ggplot2::guide_legend(override.aes = list(size=2), ncol = 1))

plt
```

Next, let’s redefine the labels of cells that are part of the subset
“C\_near\_B” using `selectLabels()`. Then, let’s specifically
visualize cells of cell type “C”, “B”, and also the cells are are
“C\_near\_B”

```
## make a temporary cell type annotation factor with labeling cells that are of a specific subset
annots_temp <- crawdad::selectLabels(df = cells,
                                     com = cells$celltypes,
                                     subset_list = subset.list,
                                     cellIDs = c("A", "B", "C", "D"), ## still keep labels for these cell types
                                     subsetIDs = c("C_near_B")) ## specifically label cells that are defined as this subset in `subset.list`

## finally, for clarity sake, we'll also rename the "C" cells that are not part of the "C_near_B"
annots_temp <- dplyr::recode_factor(annots_temp, "C" = "the other Cs")

## visualize the subset only
plt <- crawdad::vizAllClusters(cells = cells,
                               coms = annots_temp,
                               ofInterest = c("B", "the other Cs", "C_near_B"),
                               title = "All B and C cells, and the C_near_B subset",
                               axisAdj = 1, s = 6, a = 0.5) +
  ggplot2::guides(colour = ggplot2::guide_legend(override.aes = list(size=2), ncol = 1))

plt
```

## Visualize neighboring cells

To see the neighbors around our subset cells of interest and to get a
visual sense of how these neighbors influenced the subset assignment, we
can first select cells that are neighbors with a set of reference
cells.

We can define the `reference.ids` as the cells that are
part of subset “C\_near\_B” but this can be any set of cell IDs we want to
assess neighbors for.

```
neighCells <- crawdad::getNeighbors(cells = cells,
                                   reference.ids = subset.list[["C_near_B"]],
                                   removeRef = TRUE, ## whether to keep the reference cells in the output or to remove them and just look at the neighbors.
                                   dist = 100,
                                   returnSP = FALSE)

## setting `returnSP = FALSE` returns a factor of all the cells, but non-neighbors are now labeled as NA
head(neighCells)
```

Now lets use this new factor of celltype labels to visualize the
neighbors and the subset cells of interest:

```
## now let's again label the cells that are the specific subset, but using the new "neighCells" factor of celltype labels
annots_temp <- crawdad::selectLabels(df = cells,
                                     com = neighCells,
                                     subset_list = subset.list,
                                     cellIDs = c("A", "B", "C", "D"), ## original cell IDs in com of interest
                                     subsetIDs = c("C_near_B") ## subsets in subset_list of interest
                                     )

## visualize the new data.frame with the temporary annotations that specifically labels the neighbor cells and the selected subset
plt <- crawdad::vizAllClusters(cells = cells,
                               coms = annots_temp,
                               title = "C_near_B and their neighbors within dist=100",
                               axisAdj = 1, s = 6, a = 0.5) +
  ggplot2::guides(colour = ggplot2::guide_legend(override.aes = list(size=2), ncol = 1))

plt
```

Alternatively, we can choose to skip
`crawdad::selectLabels` and just visualize the neighbor cells
using the new factor of celltype labels instead of making a temporary
`annots_temp` factor like what was done above.

```
plt <- crawdad::vizAllClusters(cells = cells,
                               coms = neighCells,
                               title = "Just the neighbors",
                               axisAdj = 1, s = 6, a = 0.5) +
  ggplot2::guides(colour = ggplot2::guide_legend(override.aes = list(size=2), ncol = 1))

plt
```

This can speed up the process of finding a neighbor distance that
might be appropriate. For example, continuing to use the “C\_near\_B”
subset as our reference:

```
## control this variable `neigh.dist` to see what type of neighbor distance is appropriate
neigh.dist <- 50
neighCells <- crawdad::getNeighbors(cells = cells,
                                   reference.ids = subset.list[["C_near_B"]], ## can be any set of cells
                                   dist = neigh.dist)

plt <- crawdad::vizAllClusters(cells = cells,
                               coms = neighCells,
                               title = paste0("Neighbors with neigh.dist = ", neigh.dist),
                               axisAdj = 1, s = 6, a = 0.5) +
  ggplot2::guides(colour = ggplot2::guide_legend(override.aes = list(size=2), ncol = 1))

plt
```

## Run analysis on subsets

We can also analyze cell type colocalization patterns for the defined
subsets. For this, we just need to pass in the `subset.list`
of subsets to `findTrends()`. When using
`subset.list` in `findTrends()`, the subset.list
subsets are treated as the reference cell types and the cell types in
the `celltypes` column in `cells` are the neighbor
cell types.

(Note that within this list, we can define any set of cells for which
we would like to compute colocalization trends with respect to the cell
types in the `celltypes` column in `cells`. This
also means that we have flexibility to assess relationships between any
group of cells by changing the cell type labels in
`celltypes` and/or creating a list of specific subsets of
cells for the `subset.list`.)

```
oldw <- getOption("warn")
options(warn = -1)

results2 <- crawdad::findTrends(cells,
                        dist = 100,
                        shuffle.list = shuffle.list,
                        subset.list = subset.list,
                        ncores = ncores,
                        verbose = TRUE)
```

```
## Evaluating significance for each cell type

## using neighbor distance of 100

## Calculating trends for each subset in `subset.list` with respect to the cell types in `cells$celltypes`

## A_near_A

## B_near_A

## C_near_A

## D_near_A

## A_near_B

## B_near_B

## C_near_B

## D_near_B

## A_near_C

## B_near_C

## C_near_C

## D_near_C

## A_near_D

## B_near_D

## C_near_D

## D_near_D

## Time was 1.51 mins
```

```
options(warn = oldw)
```

```
plt <- crawdad::vizTrends.heatmap(dat = results2)
```

```
## results detected to be a list. Melting to data.frame.
```

```
plt
```

## Visualizing cells at specific resolutions

The trends generated by `CRAWDAD` allow us to observe how
different cell types are organized with each other across different
scales. For example, Two cell types may appear to be separated at small,
micro-scale resolutions, but are significantly colocalized on a global
scale. These relationships are determined by comparing the real data to
a dataset where cell labels have been shuffled at different resolutions.
This is primarily done by partitioning the dataset into regions of a
given size, or resolution, and restricting the shuffling of labels
between cells to those that are in the same region.

To get a sense of the differences between the real data and the
shuffled data, it can be useful to visualize the shuffled cells at a
particular resolution. This can be done by extracting the shuffled cell
labels at a given resolution:

```
## list hierarchy is: shuffle.list$resolution$permutation
shuff <- shuffle.list$`200`$`1`
head(shuff)

## for visualization purposes, we can also add the shuffled labels to a new column in `cells`
cells$shuff_200 <- shuffle.list$`200`$`1`

cells
```

Additionally, it may also be useful to visualize cells that are in a
specific region of the dataset. For example, maybe a cell type
colocalizes with another cell type at a more macro-scale resolution, but
only in a particular location of the tissue.

Because `cells` is an
`sp::SpatialPointDataSet()`, it is compatible with functions
from the `sf` (Simple Features) R library. One of these,
`sf::st_make_grid()`, is utilized by
`makeShuffledCells()` to define the regions for shuffling. We
can apply this function here to get the grids.

```
## shuffling grid
grid <- sf::st_make_grid(cells, cellsize = 200)

## get the coordinates of the centers of the grid tiles to add the tile IDs
grid_coords_centroids <- as.data.frame(sf::st_coordinates(sf::st_centroid(grid)))
grid_coords_centroids$name <- as.character(rownames(grid_coords_centroids))
```

```
plt <- crawdad::vizAllClusters(cells = cells,
                               coms = cells$celltypes,
                               title = "sim",
                               axisAdj = 1, s = 6, a = 0.5) +
  ggplot2::guides(colour = ggplot2::guide_legend(override.aes = list(size=2), ncol = 1)) +
  
  ## add in the grid information on top of the plot
  ggplot2::geom_sf(data = grid, fill = NA) +
  ggplot2::geom_text(data = grid_coords_centroids, ggplot2::aes(X, Y, label = name))
```

```
## Coordinate system already present. Adding new coordinate system, which will replace
## the existing one.
```

```
plt
```

```
## and here is the shuffled data:
plt <- crawdad::vizAllClusters(cells = cells,
                               coms = cells$shuff_200,
                               title = "sim shuffled at 200",
                               axisAdj = 1, s = 6, a = 0.5) +
  ggplot2::guides(colour = ggplot2::guide_legend(override.aes = list(size=2), ncol = 1)) +
  
  ## add in the grid information on top of the plot
  ggplot2::geom_sf(data = grid, fill = NA) +
  ggplot2::geom_text(data = grid_coords_centroids, ggplot2::aes(X, Y, label = name))
```

```
## Coordinate system already present. Adding new coordinate system, which will replace
## the existing one.
```

```
plt
```

If we are interested in looking at a specific grid region, we can
find the cells that specifically intersect with it:

```
## pull out cells in specific grid regions
int <- sf::st_intersection(cells, grid[[7]])
cells2 <- cells[rownames(int),]

## grid 11 real
plt <- crawdad::vizAllClusters(cells = cells2,
                               coms = cells2$celltypes,
                               title = "grid 7",
                               axisAdj = 1, s = 10, a = 0.5) +
  ggplot2::guides(colour = ggplot2::guide_legend(override.aes = list(size=2), ncol = 1))

plt
```

```
## grid 11 shuffled
plt <- crawdad::vizAllClusters(cells = cells2,
                               coms = cells2$shuff_200,
                               title = "grid 7 shuffled at 200",
                               axisAdj = 1, s = 10, a = 0.5) +
  ggplot2::guides(colour = ggplot2::guide_legend(override.aes = list(size=2), ncol = 1))
  
plt
```
